# Asymptomatic Transmission and the Dynamics of Zika Infection

**DOI:** 10.1038/s41598-017-05013-9

**Published:** 2017-07-19

**Authors:** Seyed M. Moghadas, Affan Shoukat, Aquino L. Espindola, Rafael S. Pereira, Fatima Abdirizak, Marek Laskowski, Cecile Viboud, Gerardo Chowell

**Affiliations:** 1 Agent-Based Modelling Laboratory, York University, Toronto, Canada; 2 Departamento de Física, Instituto de Ciências Exatas - ICEx, Universidade Federal Fluminense, Volta Redonda, RJ Brazil; 3 Division of Epidemiology and Biostatistics, School of Public Health, Georgia State University, Atlanta, GA USA; 4 Division of International Epidemiology and Population Studies, Fogarty International Center, National Institutes of Health, Bethesda, MD USA

## Abstract

Following the 2013–14 outbreak in French Polynesia, the Zika virus (ZIKV) epidemic spread widely to many countries where *Aedes Aegypti* as the main transmitting vector is endemic. The lack of a second wave of ZIKV infection in most affected regions may suggest that a sufficiently high level of herd immunity was reached during the first wave. We developed an agent-based transmission model to investigate the role of asymptomatic infection on the likelihood of observing a second wave, while accounting for its relative transmissibility. We found that, as the relative transmissibility of asymptomatic infection increases, a second wave is more likely to occur, despite an increase in the attack rate during the first wave. When the reproduction number varies between 1.9 and 2.8 based on estimates for Antioquia, Colombia, the attack rate varies between 4% and 26% for a low (below 10%) effectiveness of interventions in blunting the ZIKV transmission from symptomatic cases to mosquitoes. Moreover, the fraction of cases due to sexual transmission is estimated below 4% of the cumulative incidence. Our analyses underscore the need to quantify the transmissibility of asymptomatic infections, without which the overall attack rates and the level of herd immunity cannot be accurately estimated.

## Introduction

The Zika virus (ZIKV) infection, an arbovirus from the *Flaviviridae* family, spread through the Pacific and the Americas in 2015, dampened down in April 2016 after several large outbreaks^1^. Being primarily carried by *Aedes Aegypti* and transmitted through bites of infected mosquitos^2^, the climate across Latin America enhanced the ZIKV outbreaks to more northern latitudes, including several southern parts of the United States^3, 4^. Travel related cases were identified in North America, especially in Canada without local transmission through mosquito bites^5^. As predicted^6^, ZIKV infection spread globally in the absence of countermeasures such as vaccines and prophylactic drugs, especially in countries where *Aedes* mosquitoes are endemic. As of December 2016, the ZIKV infection has been reported in 69 countries and territories globally^7^.

While primary transmitting carriers are known to be infected mosquitoes^2^, other modes of transmission have been reported, including sexual encounter^8–11^, and blood transfusion^12^. This highlights the potential significance of human-to-human transmission of ZIKV infection, especially when clinical symptoms are absent. A significant portion (up to 80%) of ZIKV infection is estimated to be asymptomatic without presenting any symptoms of clinical illness^13, 14^. However, the contribution of asymptomatic ZIKV infection to the overall disease incidence has not been quantified, which introduces substantial uncertainty into modeling studies of ZIKV transmission dynamics and control interventions. This quantification is particularly important in understanding the levels of herd immunity in the population, which can prevent large-scale outbreaks if it is sufficiently high, while sporadic cases of ZIKV infection may still occur^15^. Because of unknown levels of herd immunity generated during the 2015–2016 ZIKV outbreaks in affected countries, and because of the uncertainty about asymptomatic transmission compared to symptomatic transmission, the risk of subsequent outbreaks has not been assessed in previous studies.

We sought to investigate the likelihood of observing a second wave of ZIKV infection and estimate the cumulative attack rates for difference levels of the relative transmissibility of asymptomatic infection (compared to symptomatic infection). We evaluated several plausible scenarios by varying the contribution of symptomatic cases to infection transmission based on the effect of interventions. To this end, we developed an agent-based model of ZIKV transmission between human and mosquito populations, and used the published estimates of the reproduction number to calibrate the model^16^. Using a scaled-down population of 10,000 individuals with demographic characteristics resembling those of Colombia, one of the most Zika-affected countries in South America, we generated simulations of the daily incidence of ZIKV infection over a 2-year period. In addition to estimating the effective reproduction number of ZIKV infection at the end of first wave, we demonstrate that the occurrence of a second wave of infections depends heavily on the relative transmissibility of asymptomatic infection.

## Methods

### Agent-based model

ZIKV, like other *flaviviruses*, is transmitted to humans primarily through the bites of infectious *Aedes* mosquitoes in the subgenus *Stegomyia*, particularly *Ae. Aegypti* ^17^. We therefore developed an agent-based model to include human and mosquito agents in the chain of disease transmission. For the human population, we used the population demographics of Colombia for age and sex, and generated an *in-silico* population of 10^4^ individuals (Figure S1, Supplemental Information).

In addition to vector transmission of ZIKV, a number of cases have been reported as a result of sexual contacts^8–11^, but there is considerable uncertainty about the risk of sexual transmission^18^. An epidemiological report from the US Center for Disease Control and Prevention indicates that about 1% of Zika cases resulted from sexual contact with travellers to affected areas^19^. Previous studies^20, 21^ have omitted this route of transmission for ZIKV infection dynamics due to its low risk^22^. Here, we include the possibility of ZIKV transmission through sexual contact in the model, and consider the range of 1–5% for the risk of transmission to account for its variability. We implemented sexual contacts in a monogamous context in the population, where age, sex, and frequency of sexual encounters were drawn from their associated distributions (Supplemental Information). We assumed the same risk of ZIKV sexual transmission for infectious male and female to their susceptible partners.

#### Infection stages in human and mosquitos

The model of human infection was constructed to encapsulate several epidemiological statuses of individuals, including susceptible, infected and incubating, infectious and asymptomatic, infectious and symptomatic, and recovered. The infection model for vector population includes compartments of susceptible, infected and incubating, and infectious mosquitoes.

#### Transmission dynamics

ZIKV transmission from humans to mosquitoes occurred as a result of rejection sampling-based (Bernoulli) trials where the chance of success is defined by a transmission probability distribution^23, 24^. This probability was calculated at the time of bite from a susceptible mosquito to an infectious human by

1 $${P}_{H\to M}=1-{(1-\beta )}^{{N}_{m}}$$

where *β* is the baseline probability of transmission for symptomatic cases (calibrated to a given reproduction number), and *N*_*m*_ is the number of bites of a single mosquito to an infectious individual. We assumed the same probability of ZIKV transmission from infectious mosquitoes to susceptible humans. We considered the relative transmissibility of asymptomatic infection compared to symptomatic infection as a reduction factor in *β*.

For the sexual transmission of ZIKV, we considered the probability

2 $${P}_{H\to H}=1-(1-{\beta }_{{\rm{sex}}})$$

where *β*_sex_ is the risk of sexual transmission per sexual encounter. If a case of ZIKV infection was at least 15 years old and had a sexual partner, we used rejection sampling-based trials for each sexual contact where the weekly frequency of contacts with the susceptible partner was sampled from age- and sex-dependent distributions^25, 26^, with a maximum of one encounter per day (Supplementary Information).

The effect of interventions to reduce the number of mosquito bites was included in the model as a reduction factor in *β*. We also considered the effect of condom use for symptomatic infection as a reduction factor in the risk of sexual transmission *β*_sex_.

Infected individuals experience an intrinsic incubation period (IIP) before becoming infectious^27, 28^. A fraction of infected individuals experience asymptomatic infection without developing clinical symptoms^13, 14, 28^. We assumed that recovered individuals from both asymptomatic and symptomatic infections are immune against reinfection. We assumed that infected mosquitoes have an extrinsic incubation period (EIP)^29^. Once EIP has elapsed, infected mosquitoes become infectious and remain infectious for the remaining duration of their lifetime.

### Parameterization

#### Mosquito lifespan and infection parameters

Due to similarities between Zika and dengue infections, being primarily transmitted through the bites of infectious *Aedes Aegypti* mosquitoes, we relied on parameter estimates reported in the literature for dengue infection. We assumed that mosquitoes have a lifespan determined by a hazard function given by^30^

3 $$H(t)=\frac{a{e}^{bt}}{1+\frac{as}{b}({e}^{bt}-1)}$$

For the season with a high temperature, the lifetime of mosquitoes was sampled from a discretized distribution generated by hazard and survival functions with *a* = 0.0018, *b* = 0.3228, and *s* = 2.1460, having the mean of 19.6 days^30^. The longevity of mosquitoes for the season with a low temperature was sampled from the distribution generated using *a* = 0.0018, *b* = 0.8496, and *s* = 4.2920, with the mean of 11.2 days (see Supplementary Information). All deaths in the mosquito population were replaced, thus maintaining a constant population size. The mosquito bites were implemented as a Poisson process, with a biting rate of 0.5 per day within the reported range 0.33–1 in previous studies^31–33^. This corresponds to an average of 1 bite every 2 days. We then considered the half-life of a single mosquito as the mean of a Poisson distribution, from which the number of bites was sampled. Bites for each mosquito were randomly distributed over the mosquito lifetime, with a maximum of 1 bite per day. For a bite through which a mosquito was infected, the EIP was sampled from a log-normal distribution with the shape and scale parameters of 2.28 and 0.21, and mean of 10 days (95% CI: 7, 14)^29^.

#### Human infection parameters

We sampled the IIP for an infected individual from a log-normal distribution with the shape and scale parameters of 1.72 and 0.21, and mean of 5.7 days (95% CI: 4, 8)^27^. The infectious period was also sampled from a log-normal distribution with the shape and scale parameters of 1.54 and 0.12, and mean of 4.7 days (95% CI: 3.8, 5.7)^34^. We assumed 40–80% of infected individuals experience asymptomatic infection^13, 14^.

### Model calibration and simulations

We calibrated the model to determine the baseline transmission probability *β* based on the estimated reproduction number *R*_0_ reported in our previous study^16^, ^35^. For Antioquia, Colombia, these estimates were in the range 1.9–2.8 (95% CI) with the mean of 2.2^16^. The abundance of mosquito, considered as the ratio of mosquito population to human population (*ρ*), was varied from low to high in the range 2–10. Using the calibrated transmission probability, simulations were seeded with 1 latent infection at the incubating disease state, and a fully susceptible mosquito population. All simulations started at the onset of a high-temperature season, for which the mosquito lifetime was sampled from the distribution with the mean of 19.6 days. After 26 weeks of model simulations, the lifetime was sampled from the distribution with the mean of 11.2 days for the low temperature season. This pattern was repeated for simulations over 2 years. Simulations were averaged over 2000 independent realizations for each scenario.

### Attack rates and effective reproduction numbers

Cumulative daily case incidence was calculated for each scenario and averaged over 2000 independent realizations to estimate attack rates during an outbreak. We defined an outbreak to unfold if the cumulative incidence of infection during the third disease generation interval was greater than *R*_0_, which confers a sustained transmission within the first two disease generation intervals with *R*_0_ exceeding 1. Simulations in which the disease ceased after the third generation interval were excluded in estimating the attack rate. We assumed a gamma distribution for the generation interval with the mean of 14 days and standard deviation of 2 days^36^. To calculate the probability of a second wave, we considered the fraction of simulations that resulted in an outbreak in year 2 following the outbreak in the first year. The effective reproduction number (*R*_eff_) at the end of an outbreak was estimated using the formulae

4 $${R}_{{\rm{eff}}}=(\frac{{\rm{no}}.\,\mathrm{of}\,\mathrm{susceptibles}\,\mathrm{at}\,\mathrm{the}\,\mathrm{end}\,\mathrm{of}\,\mathrm{outbreak}}{{\rm{no}}.\,\mathrm{of}\,\mathrm{susceptibles}\,\mathrm{at}\,\mathrm{the}\,\mathrm{start}\,\mathrm{of}\,\mathrm{outbreak}}){R}_{0}$$

### Simulation scenarios

Simulations were run to obtain the daily case incidence of ZIKV infection for calibrated scenarios, corresponding to *R*_0_ = 1.9, 2.2, 2.8 within the 95% confidence interval estimated for the reproduction number of ZIKV transmission in Antioquia department, Colombia^16^. For each scenario, we considered disease spread when the contribution of symptomatic ZIKV infection to disease transmission through mosquitoes was reduced by 10%, 30% and 50%. The reduction of ZIKV transmission was implemented probabilistically for each mosquito bite when the infectious case was symptomatic. Recent evaluation suggests that symptomatic cases reached levels of molecular viral load that were significantly higher than asymptomatic cases^37^. Although this level depends on the time of sampling, it may be an indication of lower transmissibility of asymptomatic infection. We therefore considered scenarios in which the reduction factor in the transmissibility of asymptomatic infection compared with symptomatic infection was in the range 0.1–0.9.

## Results

**Figure 1:**
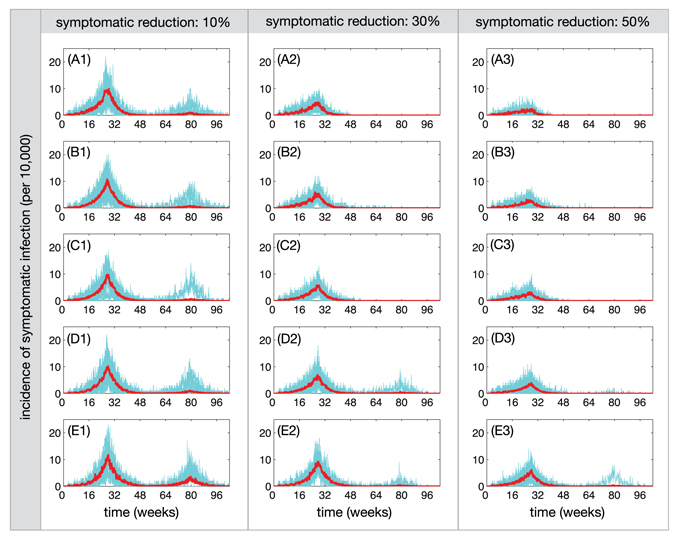
Incidence of symptomatic ZIKV infection over a 2-year period for the first and second waves, when the contribution of symptomatic ZIKV infection to disease transmission through mosquitoes was reduced by 10% (A1–E1), 30% (A2–E2), and 50% (A3–E3). The relative transmissibility of asymptomatic infection is 10% (A1,A2,A3), 30% (B1,B2,B3), 50% (C1,C2,C3), 70% (D1,D2,D3) and 90% (E1,E2,E3). The red curve represents the average of sample realizations for incidence curves.

**Figure 2:**
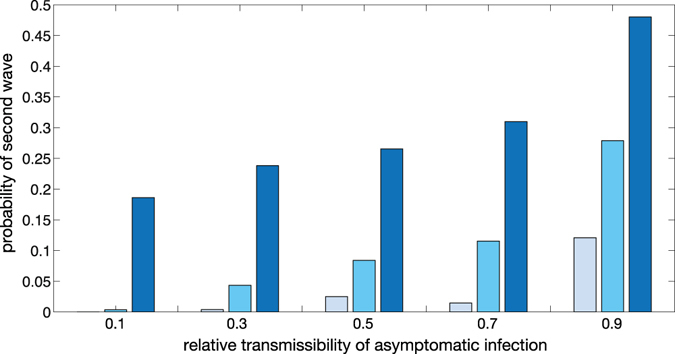
The probability of a second wave of ZIKV outbreak occurring as a function of the relative transmissibility of asymptomatic infection. Color bars correspond to scenarios in which infection transmission from symptomatic cases to mosquitoes was reduced by 10% (dark blue), 30% (light blue), and 50% (grey).

For *R*_0_ = 2.2, Fig. 1 shows the daily incidence of symptomatic infection over a 2-year period for various levels of the relative transmissibility of asymptomatic infection and different reduction levels of ZIKV transmission from symptomatic cases to mosquitoes. As the contribution of symptomatic infection to disease spread reduces (due to interventions or behavioural changes), the occurrence of a second wave of outbreak requires a higher level of the relative transmissibility of asymptomatic infection (Fig. 1). The probability of a second wave of infection occurring as a function of the relative transmissibility of asymptomatic infection is illustrated in Fig. 2. With 10% reduction of transmission from symptomatic cases, the probability of a second wave occurring increase from 0.19 to 0.48 when the relative transmissibility of asymptomatic infection increases from 10% to 90% (Fig. 2, dark blue bars). We observed the same increasing trend (with lower probabilities) for higher levels of transmission reduction from symptomatic cases (Fig. 2, light blue and grey bars).

**Figure 3:**
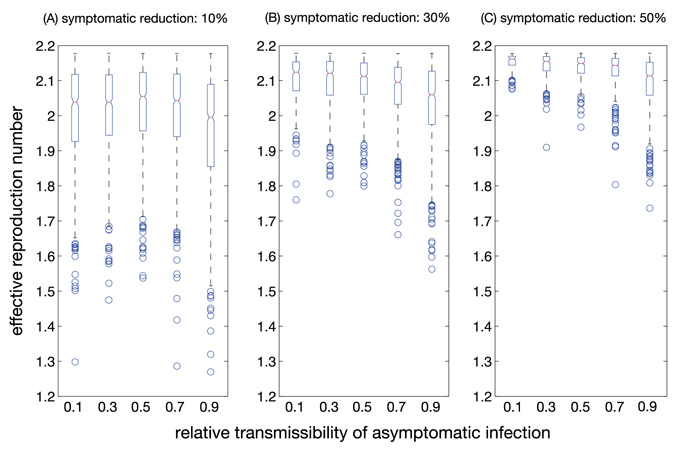
Effective reproduction number at the end of the first wave as a function of the relative transmissibility of asymptomatic infection. The contribution of symptomatic ZIKV infection to disease transmission through mosquitoes was reduced by 10% (**A**), 30% (**B**), and 50% (**C**).

**Figure 4:**
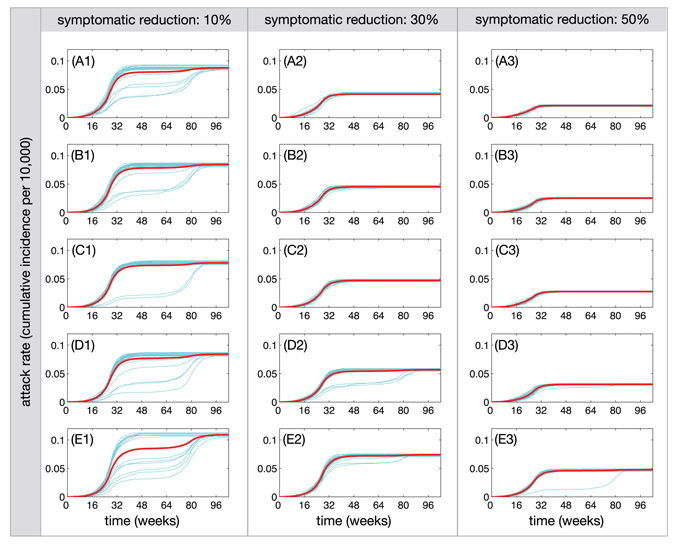
Attack rates (cumulative incidence per 10,000) of ZIKV infection over a 2-year period for the first and second waves, when the contribution of symptomatic ZIKV infection to disease transmission through mosquitoes was reduced by 10% (A1–E1), 30% (A2–E2), and 50% (A3–E3). The relative transmissibility of asymptomatic infection is 10% (A1,A2,A3), 30% (B1,B2,B3), 50% (C1,C2,C3), 70% (D1,D2,D3) and 90% (E1,E2,E3). The red curve represents the mean attack rate in each scenario within its 95% confidence interval.

For the scenarios studied here, we also estimated the effective reproduction numbers (*R*_eff_) and the cumulative attack rates at the end of the first wave of ZIKV outbreak. Figure 3 shows boxplots for the range of *R*_eff_ estimates as a function of the relative transmissibility of asymptomatic infection when *R*_0_ = 2.2 at the onset of the outbreak. With 10% reduction of ZKIV transmission from symptomatic cases, the median *R*_eff_ is 2.04 (Range: 1.64, 2.18) for 10% relative transmissibility of asymptomatic infection. The median attack rate for the corresponding scenario (Fig. 4) is estimated at 7.3% (95% CI: 6.7%, 7.7%). When the relative transmissibility increases to 90%, the median *R*_eff_ is 1.99 (Range: 1.50, 2.18), with the median attack rate of 9.3% (95% CI: 8.8%, 9.6%). With higher levels of reduction in ZIKV transmission from symptomatic cases, the median *R*_eff_ remains closer to *R*_0_ (Fig. 3), giving lower attack rates for the first wave (Fig. 4). Overall, we estimated attack rates to range from 2.2% to 11% for the scenarios simulated here with *R*_0_ = 2.2 over a 2-year period (Fig. 4). These estimates are consistent with those reported for Colombia during outbreaks through February 2017^20^.

**Figure 5:**
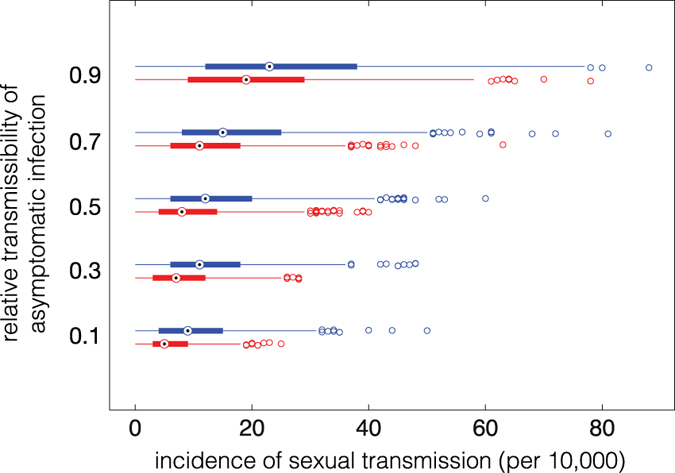
Estimated range of cumulative incidence of sexual transmission during the first wave of ZIKV infection as a function of the relative transmissibility of asymptomatic infection, in the absence of condom use (blue) and 50% condom use during symptomatic infection (red).

We estimated the cumulative number of ZIKV infection resulted from the virus transmission through sexual encounter. Figure 5 shows the range of these estimates for different relative transmissibility of asymptomatic infection in the absence of any control measure (blue bars). For a low relative transmissibility (10%), the median number of sexual transmission is 10.6 (Range: 0, 31.5), which accounts for 1.16% of the cumulative incidence (Range: 0, 2.29%). When the relative transmissibility increased to 90%, the median number of sexual transmission increased to 23 (Range: 0, 77). This corresponds to 2.4% of the cumulative incidence (Range: 1.02, 3.88%). These results suggest that the previous work in a deterministic context^38^ may have overestimated the upper bound of the fraction of cases due to sexual transmission.

We observed similar results for lower (*R*_0_ = 1.9) and higher (*R*_0_ = 2.8) reproduction numbers (Supplementary Information). These results suggest that the relative transmissibility of asymptomatic infection is a key parameter in estimating the burden of disease through different modes of transmission (i.e., mosquito bites and sexual encounter) and evaluating the probability of a second wave of ZIKV infection.

## Discussion

Our results show that the relative transmissibility of asymptomatic infection remains a key epidemiological parameter that can significantly influence disease dynamics, especially in the context of intervention strategies. We considered scenarios in which the contribution of ZIKV transmission from symptomatic cases is reduced as a result of decreased mobility and lower exposure to mosquito bites. Interventions to reduce exposure to infectious bites may include mosquito avoidance through full clothing, mosquito repellents, and spraying and larviciding. We considered the effect of interventions (regardless of their type) on the reduction of transmissibility from symptomatic cases to susceptible mosquitoes. To prevent sexual transmission of the Zika virus, condom use could be considered as an effective intervention. In an exploratory analysis, we found that the use of condoms could significantly reduce the risk of sexual transmission (Fig. 5, red bars); however, this reduction depends on the level of condom use. We observed that when interventions are absent or their effectiveness is very low in blunting the contribution of symptomatic cases to ZIKV transmission, a second wave is more likely to occur as the relative transmissibility of asymptomatic infection increases (Fig. 1). Furthermore, the occurrence of a second wave of ZIKV infection requires higher values of the relative transmissibility as the effectiveness of interventions increases (Figs 1 and 2).

The relative transmissibility of asymptomatic infection has also important implications for the use of *R*_eff_ in determining the potential for a second wave. For example, the probability of a second wave occurring is over 26% for a relative transmissibility of 0.5 when the transmission of ZIKV from symptomatic cases is reduced by 10% on average (Fig. 1, E1). In this case, the estimated *R*_eff_ has the median of 2.05 (Range: 1.71, 2.18), suggesting that the herd immunity is relatively low to prevent a second wave (Fig. 1, C1). In fact, the median attack rate is estimated at 6.6% (95% CI: 6.2%, 6.9%). However, for the same relative transmissibility, the corresponding probability for the scenario in which the transmission of ZIKV from symptomatic cases is reduced by 30% remains below 9%. In this case, while *R*_eff_ is above 1 (median: 2.11) due to the effectiveness of interventions and low attack rates of the first wave, the second wave is unlikely to occur (Fig. 1, C2). Increasing the relative transmissibility leads to higher attack rates of the first wave (Fig. 1), but may also increase the probability of a second wave unfolding (Fig. 2). These results indicate that the level of herd immunity in the population cannot be accurately measured without quantitative estimates of the contribution of asymptomatic infection.

In the context of the 2015–2016 ZIKV outbreaks in the Americas, previous work suggests that Zika spread may have contributed to the generation of herd immunity, which prevented the occurrence of a second wave of widespread ZIKV infection in the presence of sustained control efforts^15^. A recent stochastic model of ZIKV spread through the Americas estimates reporting and detection rates of 1–2%^20^. Without considering the effect of interventions or behavioural changes due to increased awareness, the model ^20^ projects a significant variation amongst attack rates in different countries, and illustrates the importance of seasonal factors in the introduction and occurrence of multiple waves of ZIKV infections. As expected^20, 39^, and shown in our simulations, these epidemic waves coincide with the seasonal pattern of mosquito lifetime. However, our results also indicate that the occurrence of a second wave depends on other factors, such as transmission reduction measures that largely influence the contribution of symptomatic infection to disease spread, and more importantly, the silent transmission of the Zika virus from asymptomatic infection. Quantifying asymptomatic transmission requires specific data on the magnitude and duration of infectiousness in infected individuals, combined with measures of exposure to biting mosquitoes during the course of infection^40^. While we do not address the riddle on the contribution of asymptomatic infection to herd immunity, our study highlights its importance in understanding the disease dynamics and the epidemiological trends observed in countries affected by the Zika virus. In a recent study, we have shown the potential for large errors that can arise in quantifying the contribution of asymptomatic infection to the overall cumulative incidence in an infectious disease outbreak^41^. These considerations call for further biological, clinical, and epidemiological studies to provide estimates of the relative transmissibility of asymptomatic infection, given its central role in determining the levels of herd immunity.

The importance of asymptomatic transmission has also been recognized in other vector-borne diseases including dengue and malaria^42, 43^. While infectiousness and severity of the disease are strongly, positively correlated with viremia^44^, outbreaks of dengue associated with low viremia have been reported^45, 46^. It has been shown that, at a given level of dengue viremia, infected individuals with no symptoms or prior to the onset of symptoms are more infectious to mosquitoes than those with symptoms^42^. In the case of Zika, asymptomatic cases with low viremia may also play a role in silent transmission of infection through sexual contacts^8–11^ and blood transfusion^12^. Within the context of previous studies^15, 20, 21, 39^, our results underscore the need to characterize and quantify the transmission potential for asymptomatic ZIKV infection. Quantitative modelling could then be used to predict the risk of infection more accurately, identify the most effective public health measures, and suggest strategies to counter vector-borne diseases with similar characteristics.

## Electronic supplementary material

Supplementary Information
