# Supplementary material for: Asymptomatic Transmission and the Dynamics of Zika Infection
Source: Sci Rep. 2017 Jul 19;7:5829. doi: 10.1038/s41598-017-05013-9 (PMC5517554; doi:10.1038/s41598-017-05013-9)
Supplement: Supplementary file 1 — Supplementary Information [file 41598_2017_5013_MOESM1_ESM.pdf]

Supplemental Information

Asymptomatic Transmission and the Dynamics of Zika Infection

Seyed M. Moghadas,[1] Affan Shoukat,[1] Aquino L. Espindola,[2] Rafael S. Pereira,[2] Fatima Abdirizak,[3] Marek Laskowski,[1] Cecile Viboud,[4] Gerardo Chowell[3,4]

[1] *Agent-Based Modelling Laboratory, York University, Toronto, Canada*

[2] *Departamento de Física, Instituto de Ciências Exatas - ICEx, Universidade Federal Fluminense, Volta Redonda, RJ, Brazil*

[3] *Division of Epidemiology and Biostatistics, School of Public Health, Georgia State University, Atlanta, GA, USA*

[4] *Division of International Epidemiology and Population Studies, Fogarty International Center, National Institutes of Health, Bethesda, MD, USA*

This supplemental information provides further details of the model, parameterization with calibration and additional simulation results.

Population demographics

We considered age and sex distributions of Colombia for a scaled down population of 10,000 individuals. These distributions are provided in Figure S1.

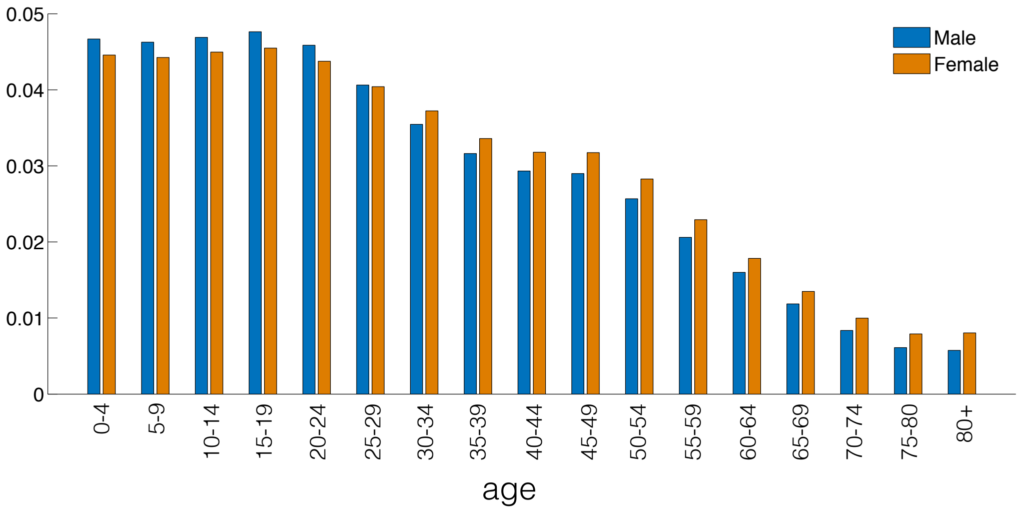

Figure S1. Age-sex distribution of the population of Colombia derived from census data [1].

Sexual encounters and partners

To implement the ZIKV sexual transmission dynamics in the model, we considered individuals above age of 15, and created partners in a monogamous context. The frequency of sexual encounters for partnered individuals was sampled from their associated distributions corresponding to sex and age of the individuals. Figure S2 represents weekly frequency of sexual encounters derived from a national probability sample among adult men and women in the United States [2,3].

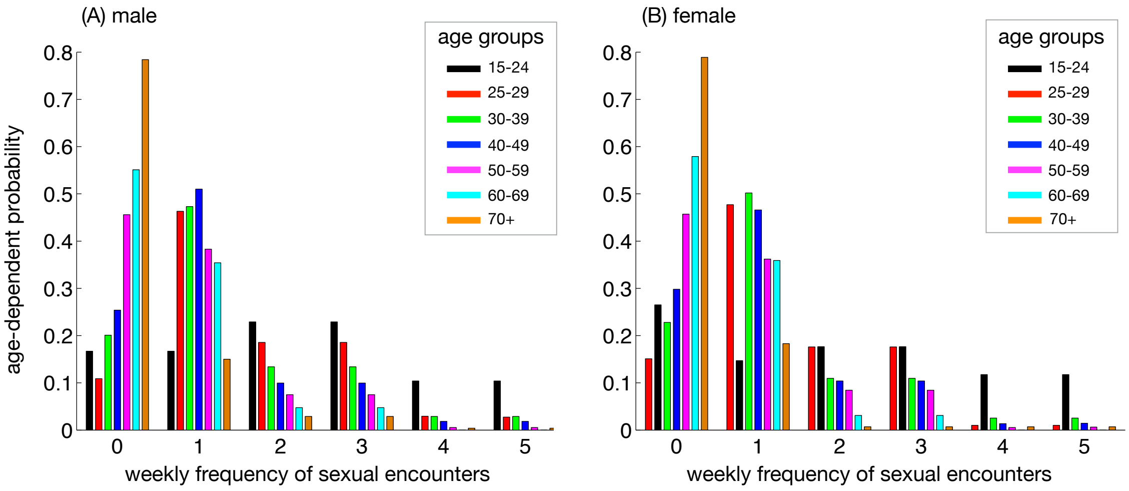

Figure S2. Age-dependent probability distributions of weekly frequency of sexual encounters among adult men and women.

Distributions of mosquito lifespan

Using the hazard (logistic function) [4], we generated the distributions of mosquito lifespan illustrated in Figure S3. The mosquito lifespan was (individually) sampled from these distributions for high- and low-temperature seasons.

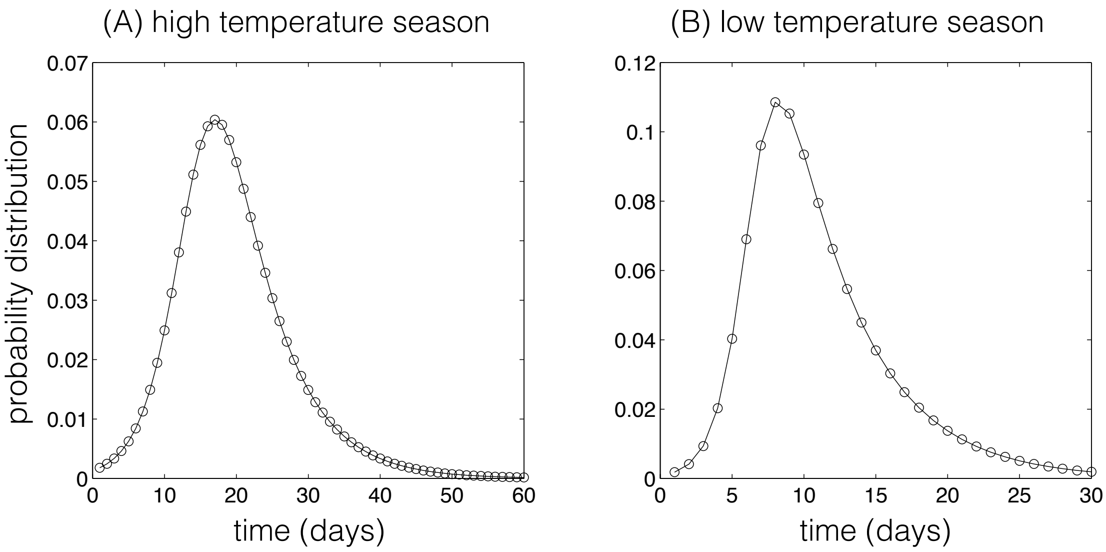

Figure S3. Distributions of mosquitos lifespan during seasons with: (A) high temperature ($a = 0.0018$, $b = 0.3228$, $s = 2.1460$) and (B) low temperature ($a = 0.0018$, $b = 0.8496$, $s = 4.2920$).

Baseline transmission probability for symptomatic infection

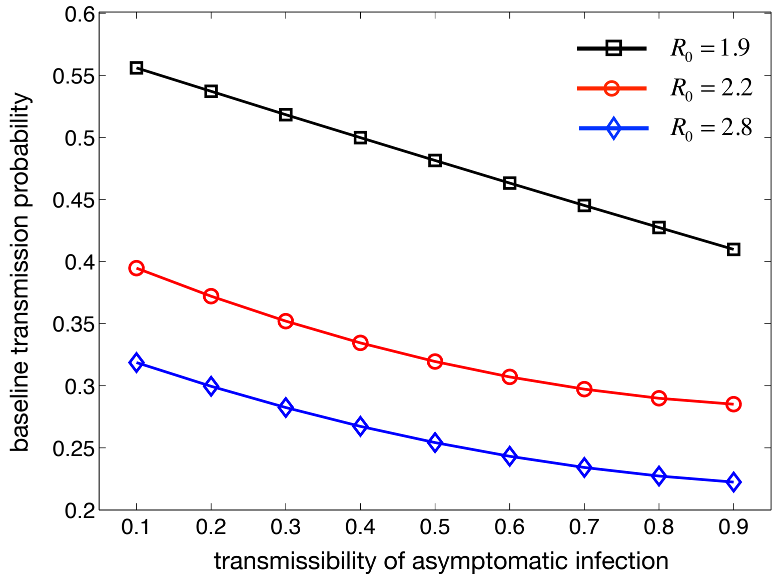

Figure S4. Calibrated probabilities of ZIKV transmission based on the reproduction number and ratio of mosquito to human populations.

Simulation Results with $R_0 = 1.9$

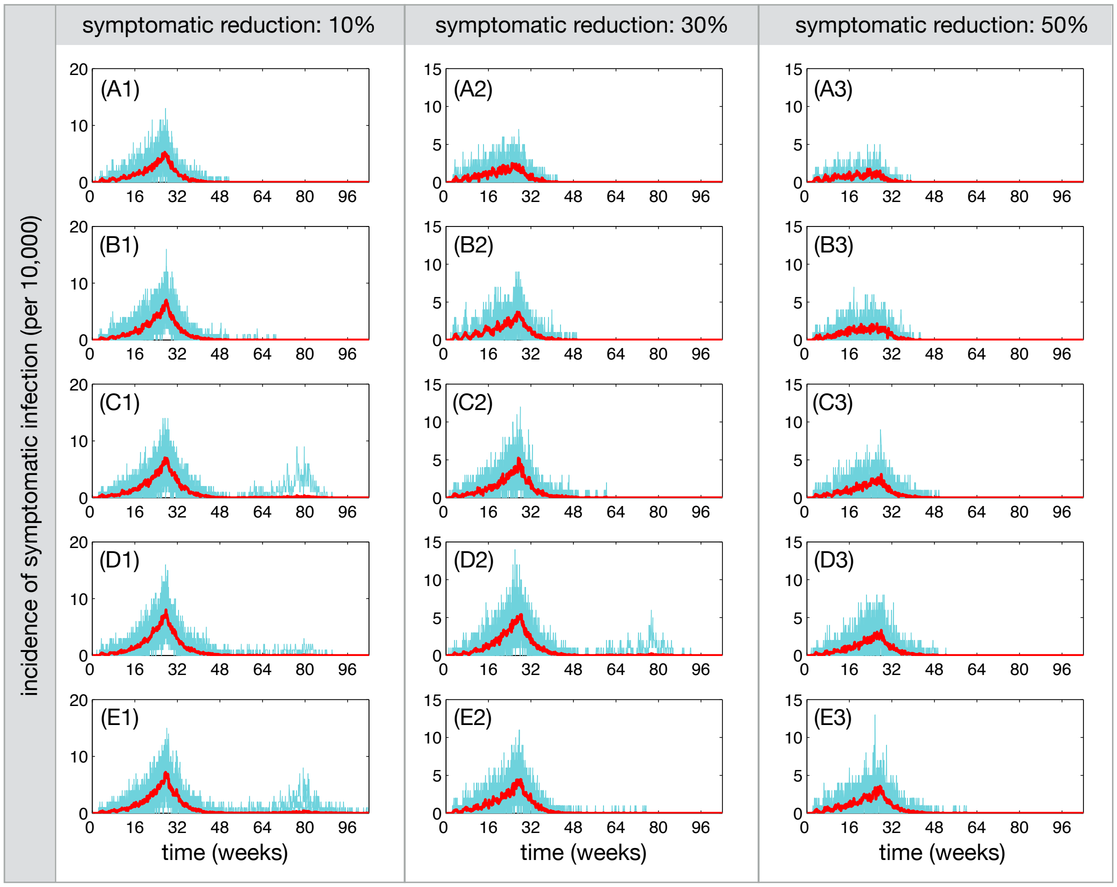

Figure S5. Incidence of symptomatic ZIKV infection over a 2-year period for the first and second waves, when the contribution of symptomatic ZIKV infection to disease transmission through mosquitoes was reduced by 10% (A1-E1), 30% (A2-E2), and 50% (A3-E3). The relative transmissibility of asymptomatic infection is 10% (A1,A2,A3), 30% (B1,B2,B3), 50% (C1,C2,C3), 70% (D1,D2,D3) and 90% (E1,E2,E3). The red curve represents the average of sample realizations for incidence curves.

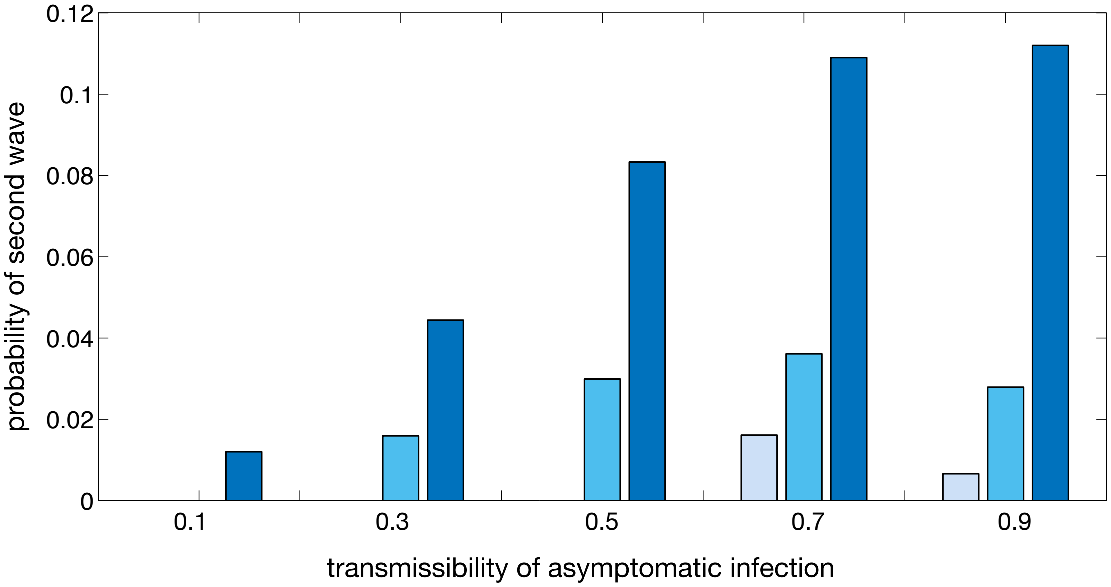

Figure S6. The probability of a second wave of ZIKV outbreak occurring as a function of the relative transmissibility of asymptomatic infection. Color bars correspond to scenarios in which infection transmission from symptomatic cases to mosquitoes was reduced by 10% (dark blue), 30% (light blue), and 50% (grey).

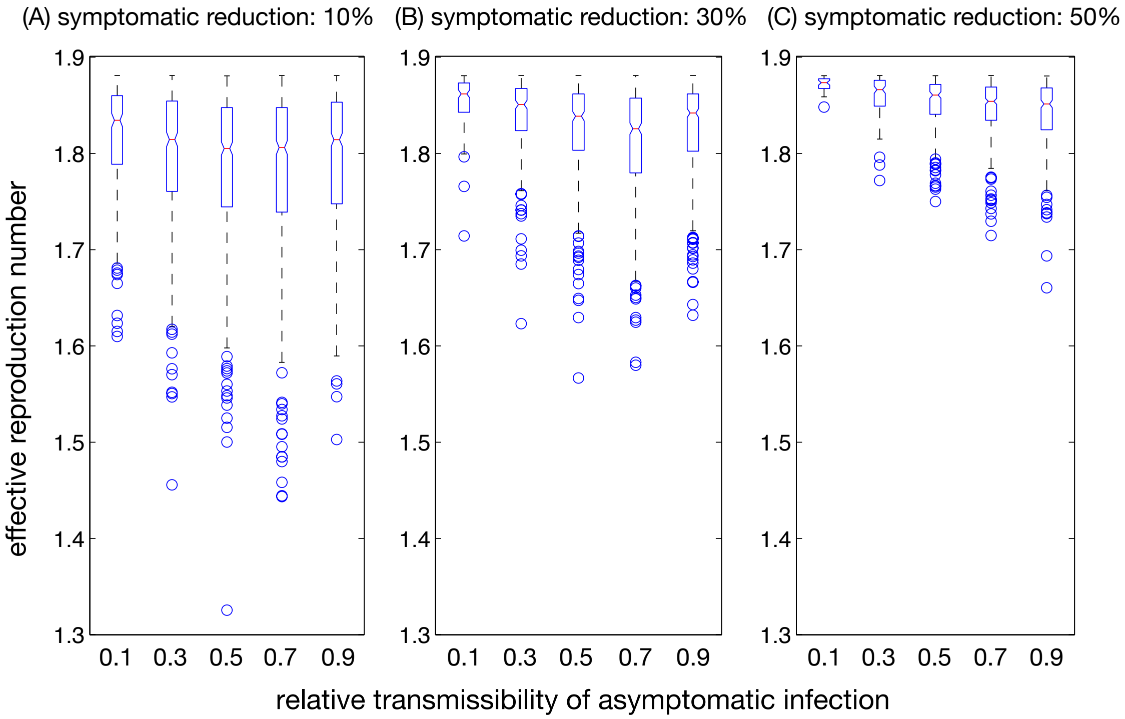

Figure S7. Effective reproduction number at the end of the first wave as a function of the relative transmissibility of asymptomatic infection. The contribution of symptomatic ZIKV infection to disease transmission through mosquitoes was reduced by 10% (A), 30% (B), and 50% (C).

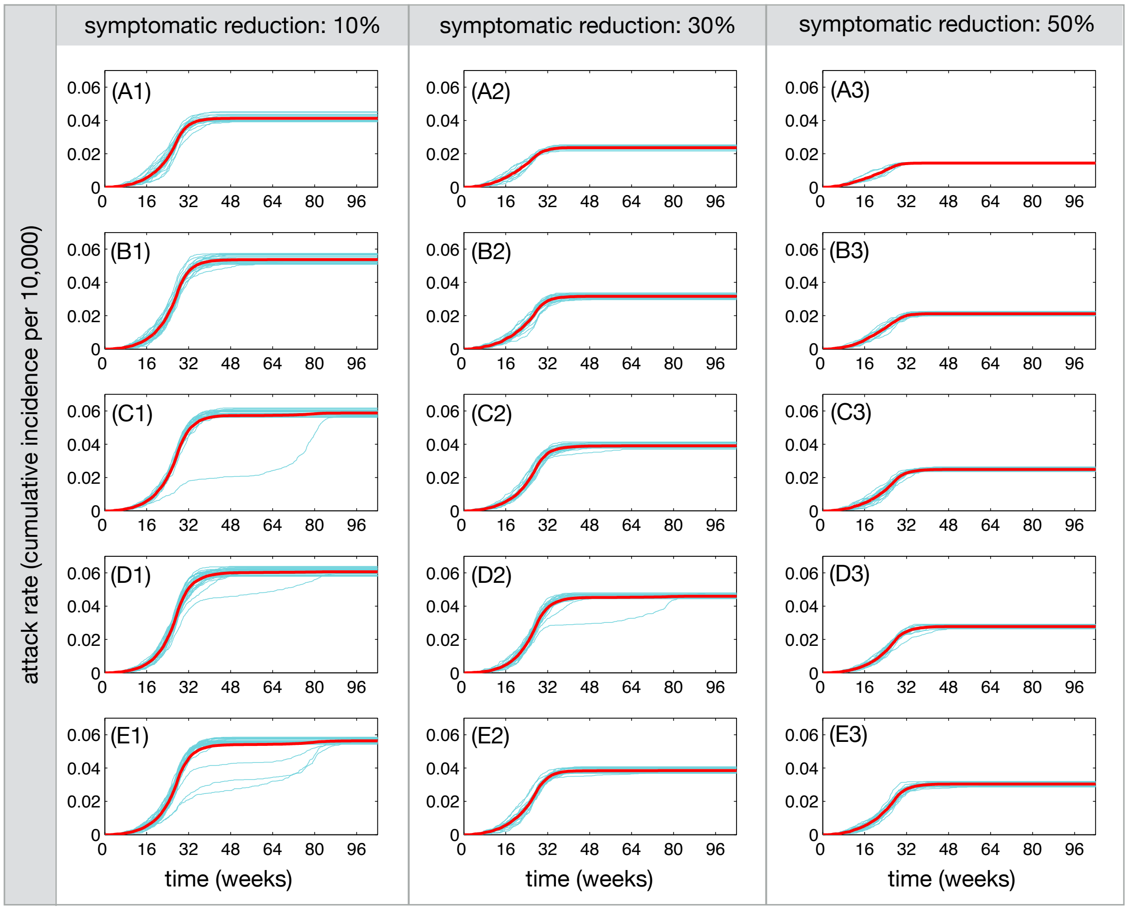

Figure S8. Attack rates (cumulative incidence per 10,000) of ZIKV infection over a 2-year period for the first and second waves, when the contribution of symptomatic ZIKV infection to disease transmission through mosquitoes was reduced by 10% (A1-E1), 30% (A2-E2), and 50% (A3-E3). The relative transmissibility of asymptomatic infection is 10% (A1,A2,A3), 30% (B1,B2,B3), 50% (C1,C2,C3), 70% (D1,D2,D3) and 90% (E1,E2,E3). The red curve represents the mean attack rate in each scenario within its 95% confidence interval.

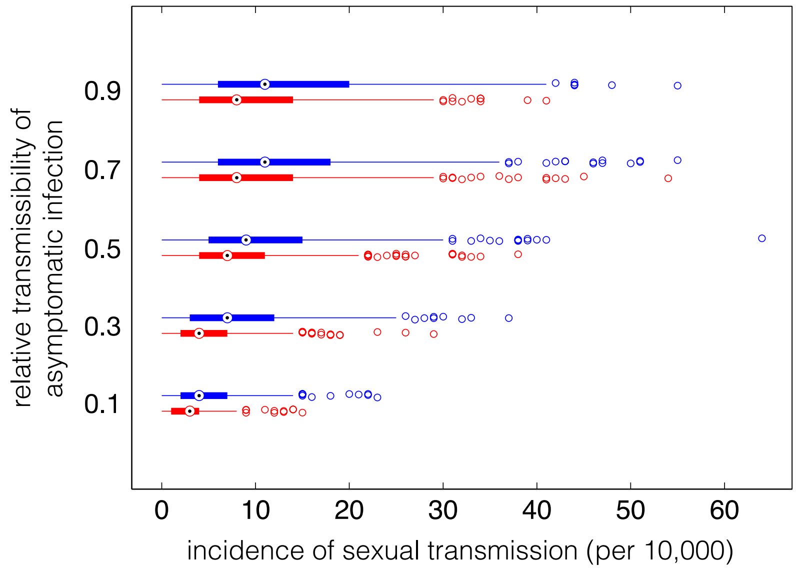

Figure S9. Estimated range of cumulative incidence of sexual transmission during the first wave of ZIKV infection as a function of the relative transmissibility of asymptomatic infection, in the absence of condom use (blue) and 50% condom use during symptomatic infection (red).

Simulation Results with $R_0 = 2.8$

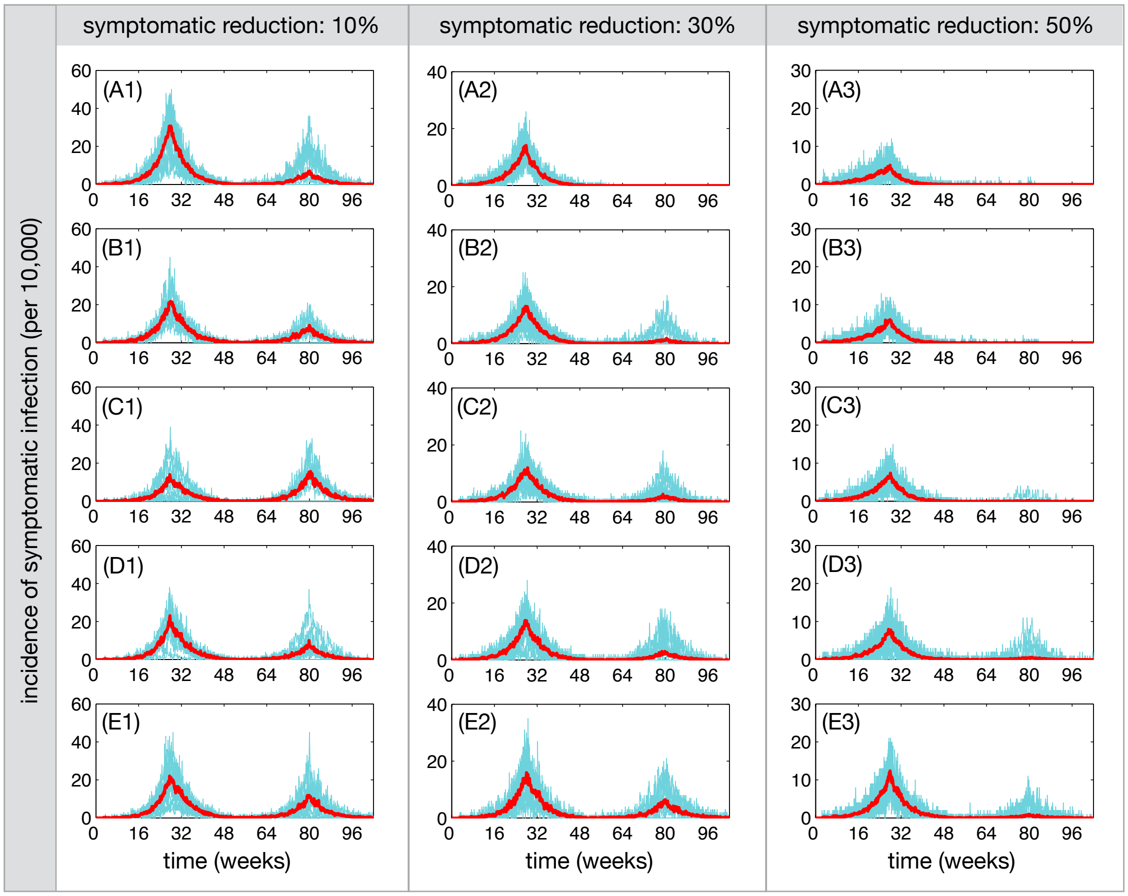

Figure S10. Incidence of symptomatic ZIKV infection over a 2-year period for the first and second waves, when the contribution of symptomatic ZIKV infection to disease transmission through mosquitoes was reduced by 10% (A1-E1), 30% (A2-E2), and 50% (A3-E3). The relative transmissibility of asymptomatic infection is 10% (A1,A2,A3), 30% (B1,B2,B3), 50% (C1,C2,C3), 70% (D1,D2,D3) and 90% (E1,E2,E3). The red curve represents the average of sample realizations for incidence curves.

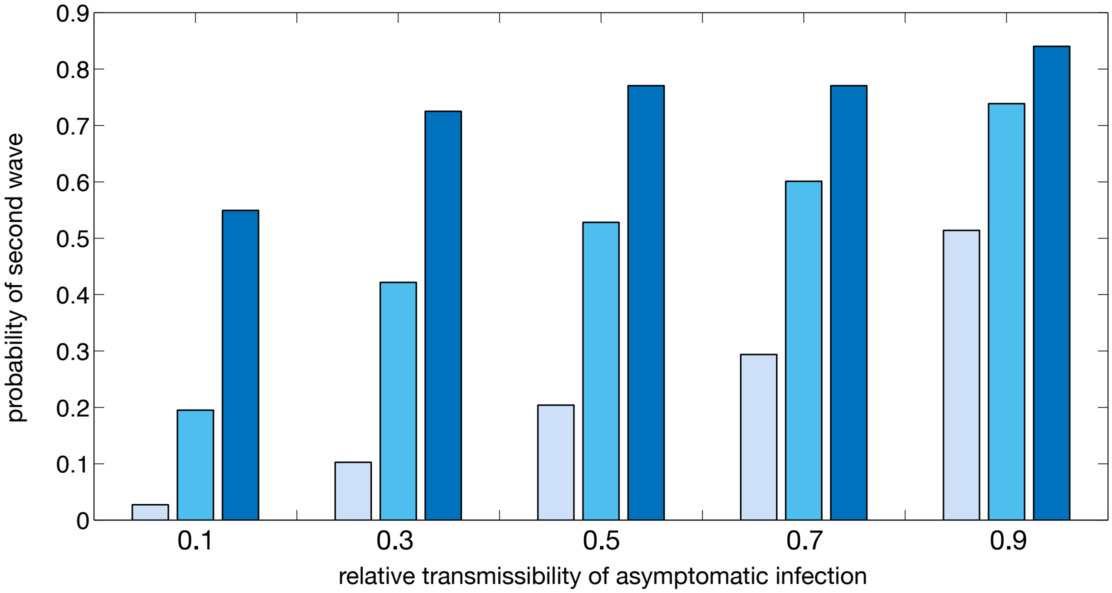

Figure S11. The probability of a second wave of ZIKV outbreak occurring as a function of the relative transmissibility of asymptomatic infection. Color bars correspond to scenarios in which infection transmission from symptomatic cases to mosquitoes was reduced by 10% (dark blue), 30% (light blue), and 50% (grey).

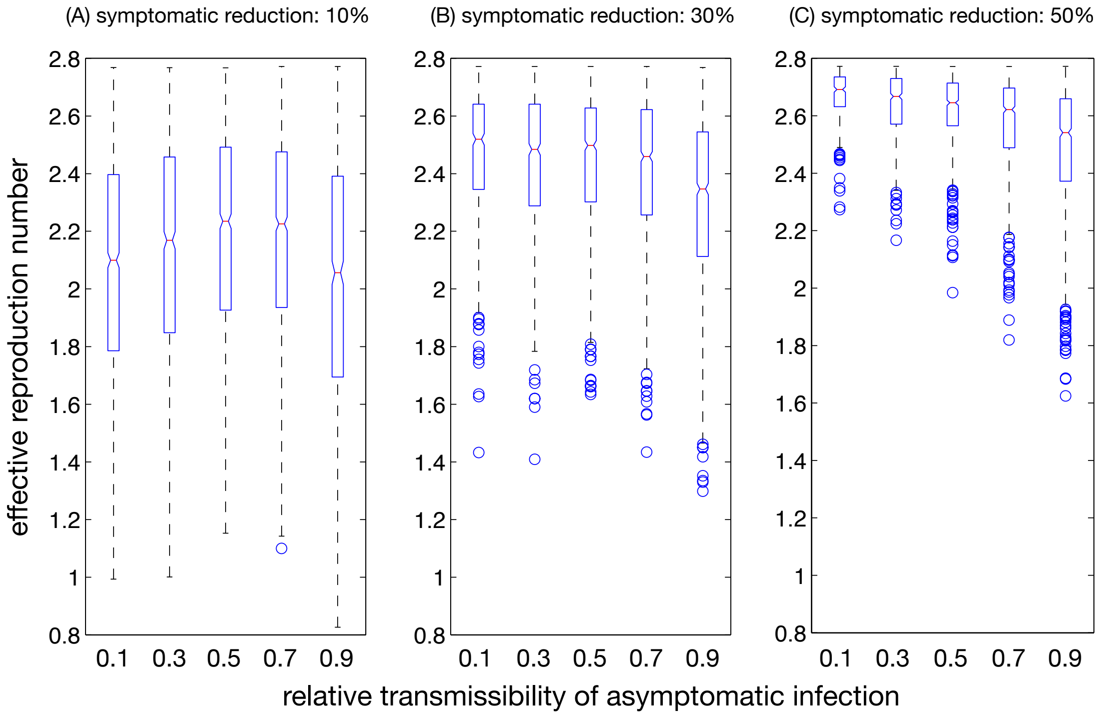

Figure S12. Effective reproduction number at the end of the first wave as a function of the relative transmissibility of asymptomatic infection. The contribution of symptomatic ZIKV infection to disease transmission through mosquitoes was reduced by 10% (A), 30% (B), and 50% (C).

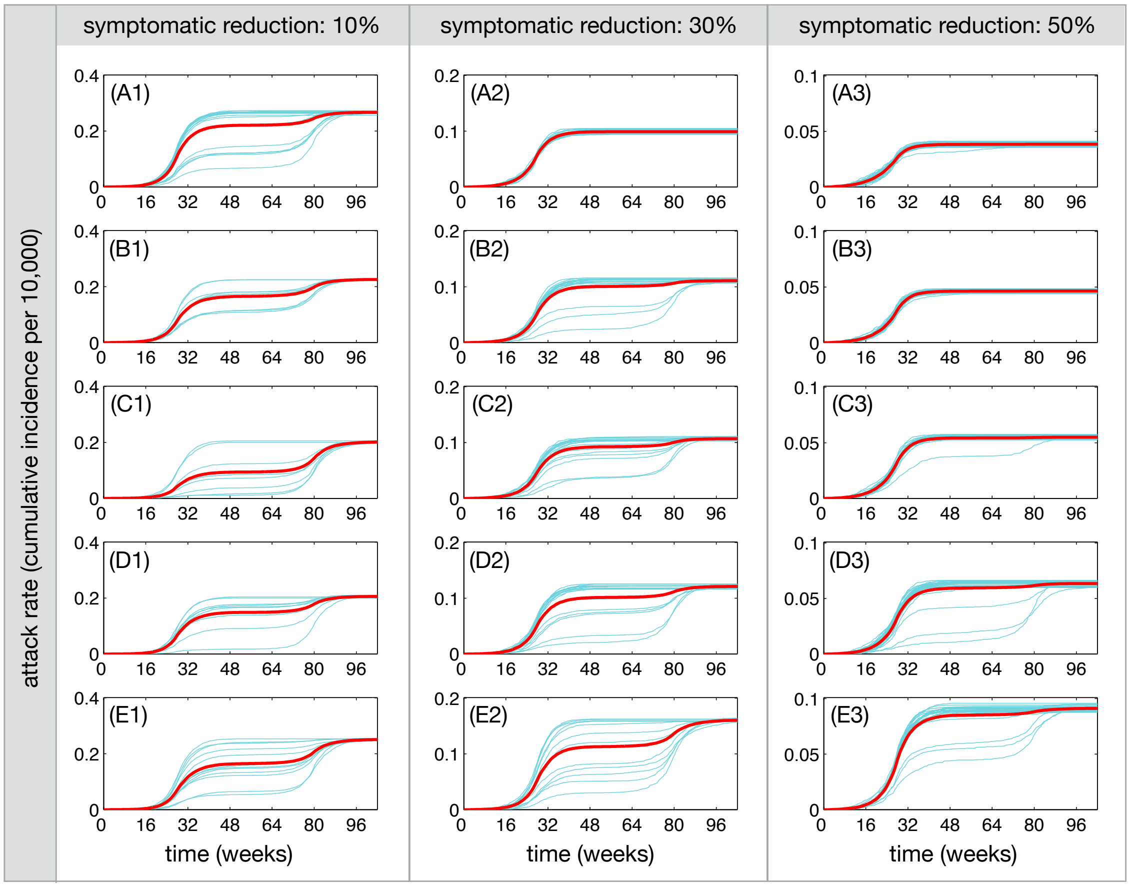

Figure S13. Attack rates (cumulative incidence per 10,000) of ZIKV infection over a 2-year period for the first and second waves, when the contribution of symptomatic ZIKV infection to disease transmission through mosquitoes was reduced by 10% (A1-E1), 30% (A2-E2), and 50% (A3-E3). The relative transmissibility of asymptomatic infection is 10% (A1,A2,A3), 30% (B1,B2,B3), 50% (C1,C2,C3), 70% (D1,D2,D3) and 90% (E1,E2,E3). The red curve represents the mean attack rate in each scenario within its 95% confidence interval.

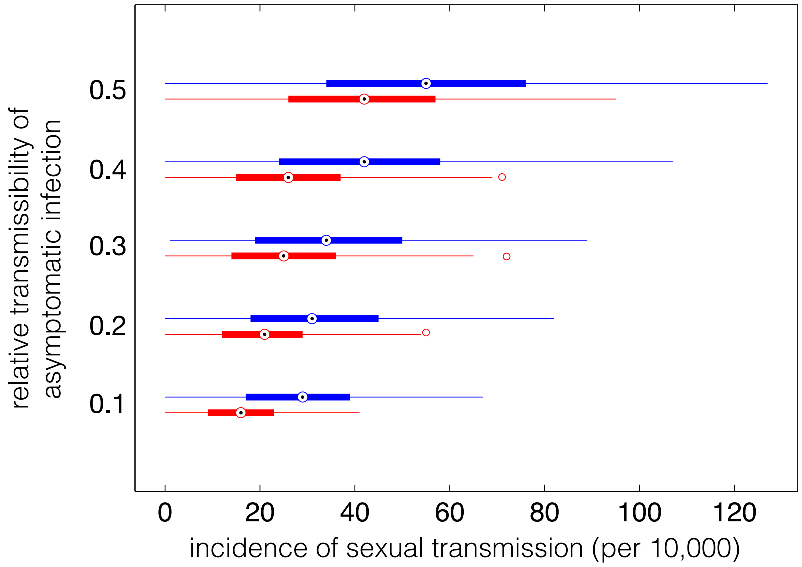

Figure S14. Estimated range of cumulative incidence of sexual transmission during the first wave of ZIKV infection as a function of the relative transmissibility of asymptomatic infection, in the absence of condom use (blue) and 50% condom use during symptomatic infection (red).

References

1. United Nations Statistics Division - Demographic and Social Statistics. http://unstats.un.org/unsd/demographic/products/dyb/dyb2.htm
2. Reece, M., Herbenick, D., Schick, V., Sanders, S.A., Dodge, B., & Fortenberry, J.D. Sexual behaviors, relationships, and perceived health among adult men in the United States: Results from a national probability sample. *J. Sex Med. 7*(s5), 291-304 (2010).
3. Herbenick, D., Reece, M., Schick, V., Sanders, S.A., Dodge, B., & Fortenberry, J.D. Sexual behaviors, relationships, and perceived health status among adult women in the United States: Results from a national probability sample. *J. Sex Med*. *7*(s5), 277-290 (2010).
4. Styer LM, Carey JR, Wang JL, Scott TW. Mosquitoes do senesce: departure from the paradigm of constant mortality. AM J Trop Med Hyg. 76:111-117 (2007).
